# Predictive role of positron emission tomography (PET) in the outcome of lymphoma patients

**DOI:** 10.1038/sj.bjc.6602040

**Published:** 2004-07-20

**Authors:** P L Zinzani, S Fanti, G Battista, M Tani, P Castellucci, V Stefoni, L Alinari, M Farsad, G Musuraca, A Gabriele, E Marchi, C Nanni, R Canini, N Monetti, M Baccarani

**Affiliations:** 1 Institute of Hematology and Medical Oncology ‘Seràgnoli’, University of Bologna, Italy; 2 Department of Nuclear Medicine, University of Bologna, Italy; 3 Department of Radiology, University of Bologna, Italy

**Keywords:** PET scan, CT scan, HD, aggressive NHL, restaging

## Abstract

An extensive analysis of the reliability of positron emission tomography (PET) after induction treatment in patients with Hodgkin's disease (HD) or aggressive non-Hodgkin's lymphoma (NHL). In all, 75 untreated patients with HD (*n*=41) or aggressive NHL (*n*=34) were studied with both PET and CT scans following standard chemotherapy induction therapy (ABVD or MACOP-B) with/without radiotherapy. Histopathological analysis was performed when considered necessary. After treatment, four out of five (80%) patients who were PET^+^/CT^−^ relapsed, as compared with zero out of 29 patients in the PET^−^/CT^−^ subset. Among the 41 CT^+^ patients, 10 out of 11 (91%) who were PET^+^ relapsed, as compared with 0 out of 30 who were PET^−^. The actuarial relapse-free survival (RFS) rates were 9 and 100% in the PET^+^ and PET^−^ subsets, respectively (*P*=0.00001). All five patients who were PET^+^/CT^−^ underwent a lymph node biopsy: in four (80%) cases, persistent lymphoma and was confirmed at histopathological examination. Two HD patients who were PET^−^/CT^+^ (with large residual masses in the mediastinum or lung) were submitted to biopsy, which in both cases revealed only fibrosis. In HD and aggressive NHL patients, PET positivity after induction treatment is highly predictive for the presence of residual disease, with significant differences being observable in terms of RFS. PET negativity at restaging strongly suggests the absence of active disease; histopathological verification is important in patients who show PET positivity.

Both Hodgkin's disease (HD) and aggressive non-Hodgkin's lymphoma (NHL) are curable malignancies. With standard induction therapies (Armitage *et al*, 1986; DeVita *et al*, 1997; Gianni *et al*, 1997) more than 70% of patients with HD and almost half those with aggressive NHL can be cured. Many prognostic indicators of response to treatment exist based on clinical characteristics at presentation. The International Prognostic Index (IPI) (The International non-Hodgkin's Lymphoma Prognostic Factors Project, 1993), which summarises different clinical prognostic factors at diagnosis, has become an established parameter for risk stratification in NHL. For HD, a specific prognostic score has been devised for the identification different risk subsets (Hasenclever and Diehl, 1998). After selecting correct, specific frontline treatments on the basis of these prognostic scores at the time of presentation, the next important steps are to: (i) distinguish between responders to standard approaches and non-responders who stand to benefit from an early change to an alternative, experimental form of therapy; (ii) monitor and identify any relapses during follow-up. Chemosensitivity and response to treatment is currently assessed on the basis of clinical, radiological and pathological (bone marrow) criteria.

X-ray computed tomography (CT) remains the gold standard for evaluation of nodal disease. However, definition of response criteria based on conventional radiographic characteristics remains difficult, as lymphoma patients treated with chemotherapy often present with residual masses of uncertain significance. These residual masses may consist of fibrotic tissue or active disease and CT scan and magnetic resonance imaging (MRI) cannot reliably differentiate between active disease and fibrosis (Hill *et al*, 1993; Zinzani *et al*, 1996). For these purposes, functional imaging with fluorine-18 fluorodeoxyglucose (FDG) positron emission tomography (PET) can reveal decisive metabolic or functional tissue parameters and is more accurate than conventional radiological imaging techniques for restaging after chemotherapy (Jerusalem *et al*, 1999, 2001; Zinzani *et al*, 1999a, 1999b; Kostakoglu and Goldsmith, 2000; Dittmann *et al*, 2001). Several groups, including our own, have evaluated the efficiency of PET for restaging patients after completed therapy (Cremerius *et al*, 1998, 2001; De Wit *et al*, 2001; Naumann *et al*, 2001; Spaepen *et al*, 2001a, 2001b; Weihrauch *et al*, 2001, 2003; Zinzani *et al*, 2002; Fihnont *et al*, 2003).

Herein, we report an extensive analysis of restaging after induction treatment of patients with HD or aggressive NHL based on PET in conjunction with CT and – in certain selected cases – histopathological analysis.

## PATIENTS AND METHODS

### Patient population

All patients with HD or aggressive NHL who were treated at our Institute with our standard induction chemotherapy protocols (with/without radiation therapy) between October 2001 and December 2002 were screened for this retrospective analysis. Eligibility criteria were no prior therapy; histological diagnosis of HD or aggressive NHL according to the R.E.A.L. classification (Harris *et al*, 1994); an ECOG (Oken *et al*, 1982) performance status score of <3; normal hepatic, renal, and cardiac function. All patients provided informed consent for the study. Ethical approval was not appropriate for this noninvasive study performed in conjunction with routine chemotherapy protocols.

### Induction treatment

Patients with HD were treated with four or six cycles of ABVD regimen (Bonadonna *et al*, 1977), based on initial staging. Patients with aggressive NHL were submitted to the MACOP-B regimen (Klimo and Connors, 1985). Radiotherapy was performed at the clinician's discretion.

### Staging

Radiological clinical staging with evaluation of tumour size included CT and PET. CT was performed at diagnosis (before therapy), 1 month after the end of chemotherapy, and 2 months after any radiotherapy. PET was performed at least 1 month after the end of chemotherapy, and 3 months after any radiotherapy. The remaining staging procedures included bone marrow biopsy, haematological and biochemical survey. A mass measuring ⩾10 cm in its longest diameter was considered as bulky.

CT scans of the chest, abdomen, and pelvis were obtained using a third generation General Electric scanner (GE Medical Systems, Milwaukee, WI, USA). All the regions were scanned at 10 × 10 mm^2^ intervals; scan time was 2 s. Intravenous contrast material (a bolus of 100–150 ml of nonionic contrast medium) was used in all patients.

To optimise FDG uptake in normal and neoplastic tissue, patients were asked to fast for at least 6 h before undergoing PET examination; no patient had a history of diabetes. Fluorine-18 fluorodeoxyglucose (FDG) was produced in our radiopharmacy using standard synthesis techniques. Each patient was i.v. injected with about 6 MBq kg^−1^ of FDG; PET scan was carried out 70–90 min after tracer injection. Before PET scanning patients were encouraged to void in order to minimise activity in the bladder. FDG-PET scans were carried out using a dedicated tomograph (Advance NX, General Electric Medical Systems, Milwakee, USA). Emission scans were acquired for 4 min at every table positron; 2-min transmission scans were also recorded in all patients. Overall, about six bed positions were required for each patient, with a total scan time of about 40 min. Images were reconstructed with segmented attenuation correction. PET images were evaluated on the basis of image visual inspection by three experienced readers. Areas of focal uptake were interpreted as positive for lymphoma unless they were at the sites of known accumulation, including the kidney and bladder, gastrointestinal tract, skeletal areas showing symmetric joint uptake (especially within the shoulder) were considered as due to arthritis.

### Evaluation of response

Complete response (CR) was defined using reported standardised guidelines (Cheson *et al*, 2000). For the purposes of this report, results of PET were not considered when assigning response status following therapy. The relapse-free survival (RFS) curve was calculated using the Kaplan–Meier method (Kaplan and Meier, 1958). RFS was defined as the period from the end of therapy to the time of first relapse. The statistical significance of differences observed were assessed using the log-rank test (Mantel and Haenzel, 1959).

## RESULTS

In total, 75 patients satisfied the eligibility criteria (Table 1

**Table 1:** Patients’ characteristics

Number of patients (HD/aggressive NHL)		75 (41/34)
Age (years)	Median	41
	Range	16–60
Sex	M	54 (72%)
	F	21 (28%)
Symptoms	No	40 (54%)
	Yes	35 (46%)
Stage	II	28 (37%)
	III–IV	47 (63%)
Bulky disease		30 (40%)

). After completion of the respective chemotherapy programs (ABVD for HD, MACOP-B for NHL), 45 out of 75 (60%) patients received radiation therapy with a tumour dose of 30–36 Gy.

Table 2

**Table 2:** PET and CT scans: restaging results and clinical outcome

	**No. pts**	**Relapses**
CT^–^/PET^–^	29	0
CT^–^/PET^+^	5	4 (80%)
CT^+^/PET^+^	11	10 (91%)
CT^+^/PET^–^	30	0

reports PET and CT restaging findings after induction with respect to outcome. After the treatment, 59 out of 75 (79%) patients were negative at PET, the remaining 16 being positive. In total, 14 of the 16 (88%) patients who were PET^+^ went on to have local relapse/progression (at a median of 9 months after completing induction therapy, range 3–12 months). By contrast, none of the 59 patients who were PET^−^ had local relapse/progression (*P*=0.00001). Figure 1

**Figure 1:**
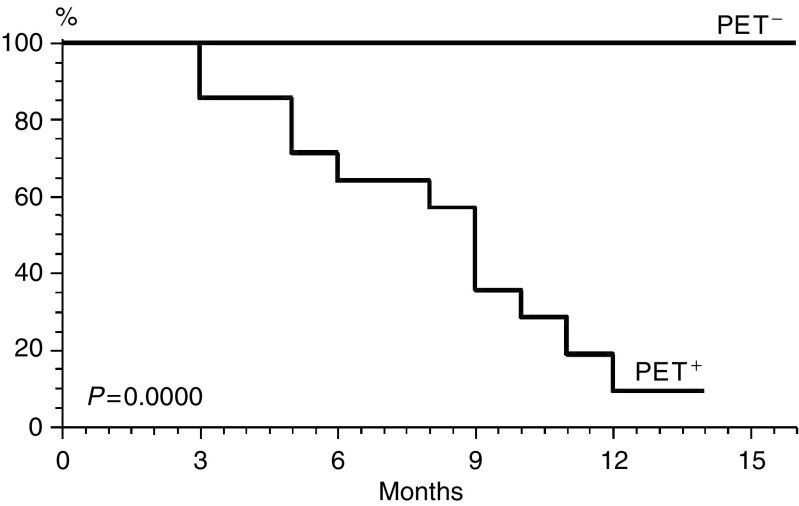
RFS curves of patients with PET negativity (*n*=59) or PET positivity (*n*=16) after induction treatment.

shows the RFS curves for patients who were PET^+^ went after the induction treatment (*P*=0.00001).

**Figure 2:**
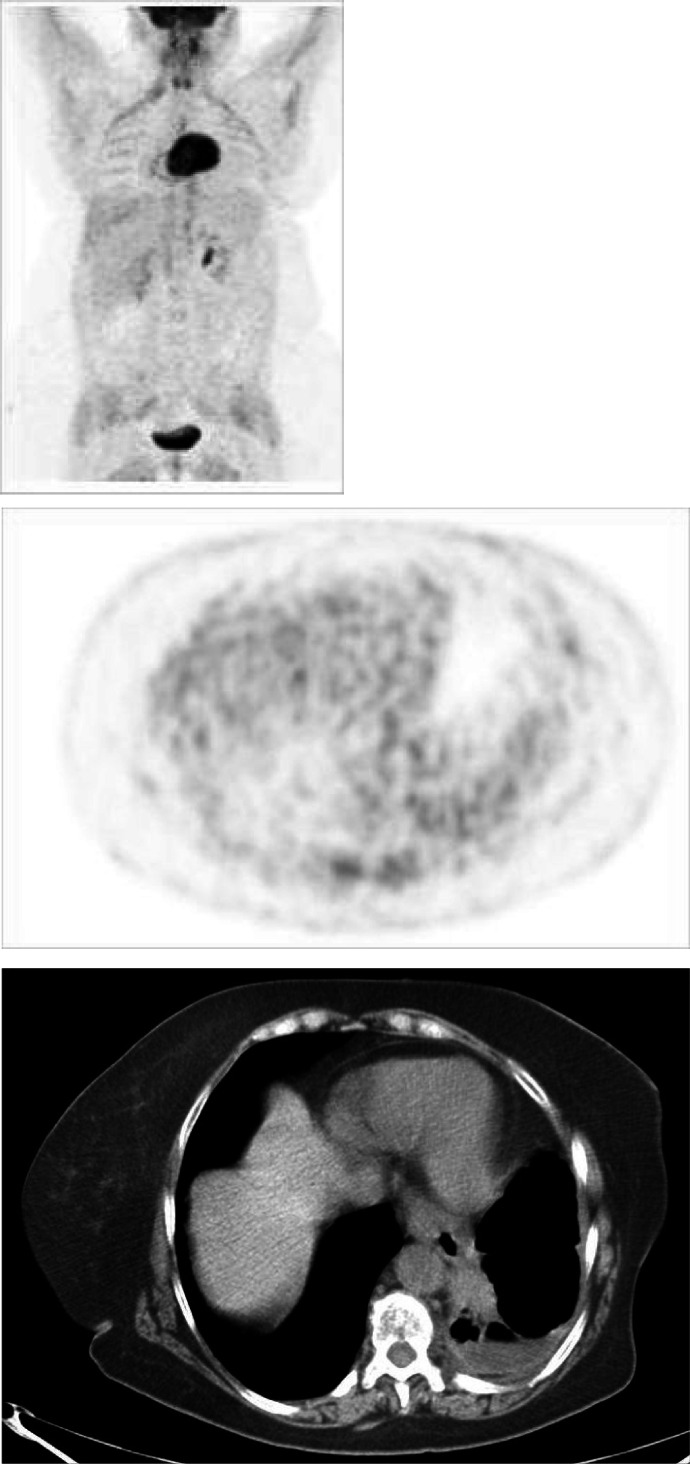
FDG PET (maximum intensity projection – MIP-image) showing no area of increased uptake. PET transaxial image shows no left lung increased uptake; corresponding transaxial CT image shows an equivocal subpleural area of consolidation in the left lower lobe. After lobectomy, this finding turned out to be benign (fibrosis and necrosis).

As regards CT, as many as 41 out of 75 (55%) patients had a positive scan. Of these, 30 out of 41 (73%) were PET^−^. All 30 (100%) patients in this PET^−^/CT^+^ subset are still in CR at a median follow-up of 12 months (range, 6–15 months). Two of these patients, both with HD, were submitted to histopathological evaluation, due to evidence at CT of either diffuse mediastinal residual disease or right lung infiltration. In both cases, the histopathological findings (based on an intrathoracic biopsy and lobectomy, respectively) were negative for lymphomatous infiltration, and revealed only fibrotic and necrotic tissue (Figure 2

shows the negative PET scan of the patient who had suspected pulmonary infiltration at CT). By contrast, in the PET^+^/CT^+^ subset 10 out of 11 (91%) patients experienced local relapse/progression within the first 12 months; in the remaining patient, a driven true-cut biopsy guided by abdominal ultrasonography was performed at 3 months (following a second positive PET scan), revealing a pathohistological diagnosis of aggressive NHL.

Among the patients who were CT^−^/PET^+^ after treatment, only one out of five (20%) is currently in CR; the remaining four (80%) patients relapsed (after 3, 5, 9, and 12 months). All these five patients were submitted to a lymph node biopsy: in the four patients who relapsed histopathological confirmation was made of either aggressive NHL (three cases) or HD (one case); in the remaining patient, the histopathological diagnosis was follicular hyperplasia. By contrast, all 29 patients in the PET^−^/CT^−^ subset are now in CR with a median follow-up of 14 months.

**Figure 3:**
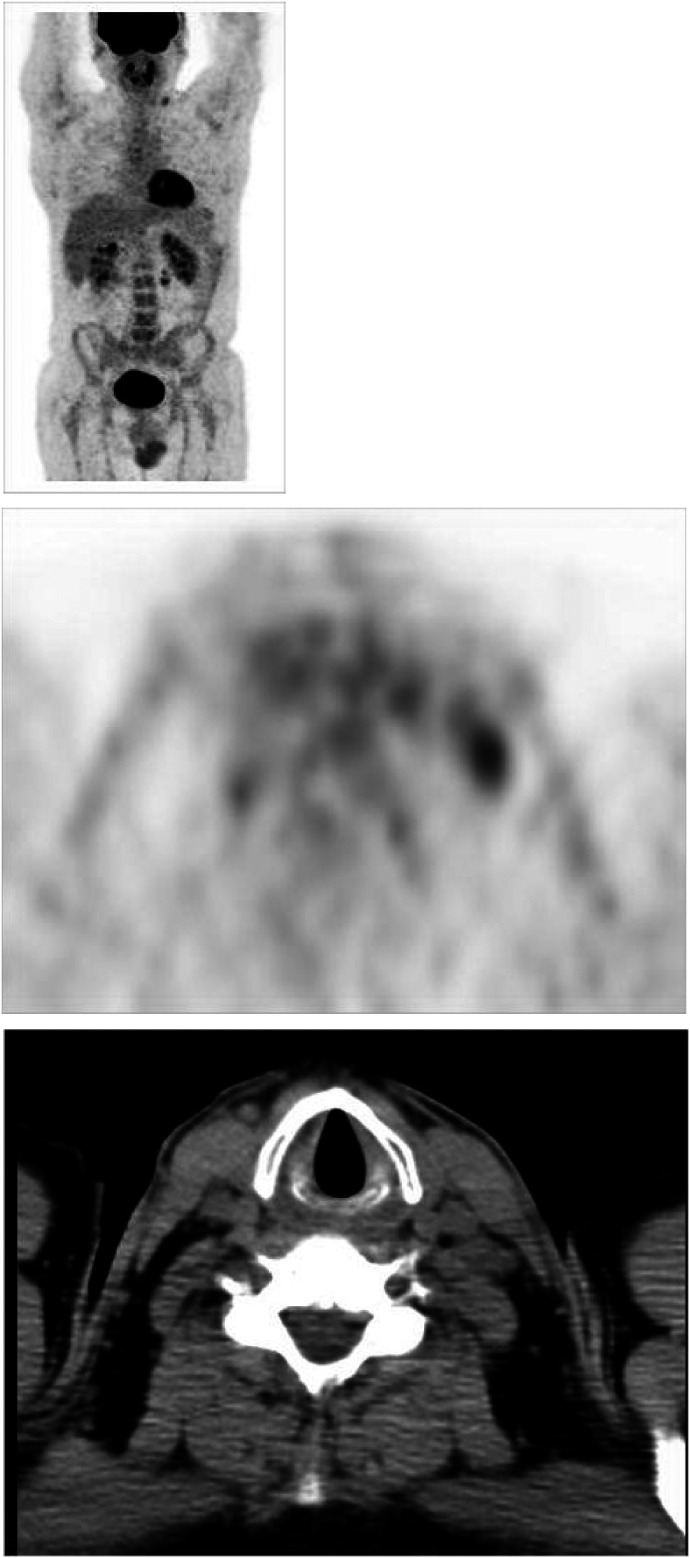
FDG PET (maximum intensity projection – MIP-image) showing an area of increased uptake in a left latero-crevical lymph node and a second area of increased uptake in abdomen, likely consistent with juxtaintestinal lymph node. Corresponding transaxial PET and CT images show the enlarged left laterocervical lymph node. Biopsy was positive for lymphoma relapse.

It should be noted that biopsies performed in two patients who were PET^−^ but strongly CT^+^ revealed only fibrotic/necrotic tissue. Figure 3

shows the positive PET scans of patient who had histopathological confirmation of relapse.

## DISCUSSION

This extended follow-up analysis strongly reinforces the positive role of PET for optimal restaging of patients with HD or aggressive NHL after induction chemotherapy with/without radiation therapy.

Many lymphoma patients present with residual masses after completing induction therapy, but less than 20% of them will eventually relapse. Thus, detection of residual active lymphoma after induction is of major clinical importance. Before the introduction of PET for clinical evaluation of lymphoma patients, no available tool could reliably predict the course of the disease. None of the morphological characteristics described for CT scan can differentiate active lymphoma from necrosis and fibrosis. MRI has a low sensitivity and is therefore not useful for lymphoma (Hill *et al*, 1993; Zinzani *et al*, 1996). Although 67-Gallium scintigraphy is a metabolic imaging technique to detect active tumour tissue, it has drawbacks such as a low spatial resolution and difficulty in identifying residual abdominal masses (Kostakoglu *et al*, 1992; Setoain *et al*, 1997; Zinzani *et al*, 1999a, 1999b). In recent years, a series of reports have shown that PET is the most helpful noninvasive metabolic imaging technique for patients with either HD or aggressive NHL: it can distinguish between active lymphoma and fibrosis, and it has important prognostic value after completion of front-line therapy (Cremerius *et al*, 1998, 2001; De Wit *et al*, 2001; Naumann *et al*, 2001; Spaepen *et al*, 2001a, 2001b; Weihrauch *et al*, 2001, 2003; Zinzani *et al*, 2002; Fihnont *et al*, 2003).

Our extensive analysis clearly indicates reliability of PET in the post-treatment evaluation of HD and aggressive NHL with respect to specific histopathological evaluation. There were no false-negative results among the 75 PET scans performed and only one false-positive result. Among the 16 patients who had positive PET scans, 14 (88%) relapsed or progressed within 1 year from the end of the treatment. Remarkably, surgical biopsy revealed persistence of active disease in four out of the five (80%) patients in whom a positivity at PET was accompanied by a negative CT scan. The single false-positive PET finding corresponded to a histolopathological diagnosis of follicular hyperplasia. Biopsies were also performed in two patients who were negative at PET due to the large size of residual masses visualised at CT: in either case, histopathological examination revealed only fibrotic/necrotic tissue. The relevant impact of PET positivity on RFS, independently of lymphoma type (HD or aggressive NHL) and presence/absence of a positive CT scan can be clearly seen from Figure 1.

Taken together, these data suggest the need to reach a histopathological diagnosis whenever possible before commencing salvage therapy, rather than relying exclusively on a positive PET result. On the other hand, the apparently strong negative predictive value of PET suggests that biopsy might be reserved for cases of PET positivity.

An interesting development in the role of PET for HD and aggressive NHL patients will be to verify and to confirm the preliminary data reported by Spaepen *et al* (2002) concerning the possible role of an early PET scan performed after a few courses of induction chemotherapy for identification and stratification of responder and nonresponder patients. This could lead to inclusion of PET in a specific mixed prognostic score including biological, clinical and imaging parameters. In addition, recent reports indicate that PET can be used to predict the clinical outcome after high-dose chemotherapy and stem cell transplantation (Becherer *et al*, 2002; Cremerius *et al*, 2002; Spaepen *et al*, 2003). In both situations, histopathological diagnosis will remain important for deciding further treatment of patients with positive PET scans.

A further step will be evaluation of the utility of combined PET/CT (Visvikis and Ell, 2003). In the last few months, we have initiated a prospective study to assess the possible predictive role of PET/CT during induction chemotherapy (after one-third and two-thirds of the courses) for patients with HD and aggressive NHL in our institutions.
